# Accumulation of Deleterious Effects in Gastric Epithelial Cells and Vascular Endothelial Cells In Vitro in the Milieu of *Helicobacter pylori* Components, 7-Ketocholesterol and Acetylsalicylic Acid

**DOI:** 10.3390/ijms23116355

**Published:** 2022-06-06

**Authors:** Adrian Ł. Gajewski, Mateusz Gawrysiak, Agnieszka Krupa, Tomasz Rechciński, Maciej Chałubiński, Weronika Gonciarz, Magdalena Chmiela

**Affiliations:** 1 Department of Immunology and Allergy, Medical University of Lodz, Pomorska 251, 92-213 Lodz, Poland; mateusz.gawrysiak@stud.umed.lodz.pl (M.G.); maciej.chalubinski@umed.lodz.pl (M.C.); 2 Department of Immunology and Infectious Biology, Faculty of Biology and Environmental Protection, Institute of Microbiology, Biotechnology and Immunology, University of Lodz, Banacha 12/16, 90-237 Lodz, Poland; agnieszka.krupa@biol.uni.lodz.pl (A.K.); weronika.gonciarz@biol.uni.lodz.pl (W.G.); 3 Department and Chair of Cardiology, Medical University of Łodz, Kniaziewicza 1/5, 91-347 Lodz, Poland; tomasz.rechcinski@umed.lodz.pl

**Keywords:** *Helicobacter pylori*, 7-ketocholesterol, acetylsalicylic acid, reactive oxygen species

## Abstract

The Gastric pathogen *Helicobacter pylori* (*HP*) may influence the development of coronary heart disease (CHD). *H. pylori* induce reactive oxygen species (ROS), which transform cholesterol to 7-ketocholesterol (7-kCh), a CHD risk factor. Acetylsalicylic acid (ASA)—an Anti-aggregation drug used in CHD patients—may increase gastric bleeding and inflammation. We examined whether *H. pylori* driven ROS effects in the cell cultures of gastric epithelial cells (AGS) and vascular endothelial cells (HUVEC) progress in the milieu of 7-kCh and ASA. Cell cultures, exposed to 7-kCh or ASA alone or pulsed with the *H. pylori* antigenic complex—Glycine acid extract (GE), urease (UreA), cytotoxin associated gene A (CagA) protein or lipopolysaccharide (LPS), alone or with 7-kCh and ASA—were examined for ROS, apoptosis, cell integrity, interleukin (IL)-8, the activation of signal transducer, the activator of transcription 3 (STAT3), and wound healing. ASA and 7-kCh alone, and particularly in conjunction with *H. pylori* components, increased the ROS level and the rate of apoptosis, which was followed by cell disintegration, the activation of STAT3, and IL-8 elevation. AGS cells were unable to undergo wound healing. The cell ROS response to *H. pylori* components may be elevated by 7-kCh and ASA.

## 1. Introduction

The role of infectious agents in the development and maintenance of atherosclerosis has been suggested for several years [1,2]. In 1994, Mendall et al. showed the elevation of specific antibodies towards the gastric pathogen *Helicobacter pylori* (*H. pylori*) in the majority of patients with coronary heart disease for the first time (CHD) [3]. Research conducted in African ethnic groups with a low incidence of classic risk factors for CHD and a high prevalence of *H. pylori* infection strengthened the argumentation for the relationship between *H. pylori* and CHD [4]. The direct interactions of these bacteria with the host cells or their influence on them via bacterial soluble components may link local *H. pylori* infection in the gastric mucosa with the development of a systemic inflammatory response [5,6]. Previously, we showed that *H. pylori* infection acts synergistically with a high fat diet in the development of proinflammatory and proatherogenic endothelial cell environments in an experimental model of *Caviae porcellus* [7]. The arteries of *H. pylori* infected animals, regardless of diet, were less elastic, suggesting the role of the infectious agent in arterial stiffness. We also showed the infiltration of inflammatory cells into the internal wall of the endothelium. Using an in vitro model of human THP-1 monocytes, we observed that *H. pylori* components, mainly proteins, induced strong foam-forming, comparable to 7-kCh, which was used as positive control [7].

Various *H. pylori* components, including vacuolating cytotoxin (VacA), urease, and lipopolysaccharide (LPS), may initiate gastric barrier disintegration [8,9,10], and drive the systemic inflammatory response via an activation of the vascular endothelium as well as immunocompetent cells [11,12]. *H. pylori*, particularly strains producing the cytotoxin associated gene A (CagA) protein, are considered pro-thrombocytic due to their elevation of the systemic concentration of thrombin—Factor VII, prothrombin subunits F1+2, fibrinogen, plasminogen, activator inhibitor type-1, and von Willebrand factor [12].

The autoimmune concept of CHD development in *H. pylori* positive individuals has also been suggested. Several *H. pylori* components, including heat shock protein B (HspB) or the Lewis X and Y determinants of LPS induce antibodies, potentially autoreactive, which due to complement activation may drive the inflammatory response in the milieu of autoantigen deposition in the gastric mucosa or vascular endothelium [13,14,15].

Oxidative stress is involved in the pathogenesis of CHD [16], as well as *H. pylori* related diseases [10]. ROS that are elevated during *H. pylori* infection may increase the level of oxidized low-density lipoprotein (oxLDL), which is a pro-atherogenic risk factor [17,18]. It was shown that oxLDL induces the transformation of macrophages into the foam cells in atherosclerotic plaques, and up-regulates the deposition of Toll-like receptors (TLR4) on macrophages, and thus induces proinflammatory signaling pathways and cell activation [19]. Furthermore, oxLDL causes apoptosis of the vascular smooth muscle cells, and downregulates the proliferation and differentiation of bone marrow endothelial progenitor cells [20]. Acetylsalicylic acid (ASA) is commonly used as an anti-inflammatory drug, and due to its anti-aggregation properties, it lowers the risk of myocardial infarction and clot-related strokes [21]. ASA protects endothelial cells via a nitric oxide/cGMP pathway. It has been shown that ASA induces beneficial defense mechanisms towards pathogenic microorganisms or the apoptosis of cancer cells on ROS dependent pathways. [22,23,24]. However, ASA’s ability to induce ROS may be detrimental to normal cells. At a high concentration, ROS may be cytotoxic to normal cells due to its damaging of DNA and its degradation of lipids, proteins, and carbohydrates [25].

The anti-platelet therapy based on ASA, recommended in CHD patients for long-term use, can cause side effects manifested by bleeding as well as gastric epithelial barrier damage and dysfunction due to ASA cytotoxicity [21,26,27]. The erosion of the gastric barrier may facilitate *H. pylori*’s colonization and penetration of *H. pylori* components into the bloodstream where they can activate leukocytes and endothelial cells or even cause vascular microinjuries. Endothelial injury, when correlated with the inhibition of cell regeneration, may expose the endothelium to chronic inflammation and thus facilitate plaque formation. It should be considered that due ASA’s cytotoxic effect, especially in the gastric epithelium [21,26,27], it may be involved in the development of an inflammatory reaction in this milieu. The activated immunocompetent cells involved in this process may provide factors intensifying oxidative stress.

In the previous study, we showed that well defined *H. pylori* soluble components, which accumulate locally in the stomach during *H. pylori* infection, generate ROS-dependent apoptosis in gastric tissue [10]. The high prevalence of *H. pylori* infections, which are chronic and frequently asymptomatic, may suggest that these bacteria can deliver components that can be spread, similarly to the antigens of gut microbiota, and thus stimulate the local and systemic inflammatory response [5].

The deleterious effects driven by *H. pylori* in the gastric mucosa can be manifested by an increased permeability of this barrier and the systemic distribution of *H. pylori* components, which in the bloodstream may stimulate vascular endothelial cells and immune cells to deliver proinflammatory cytokines and more ROS, and thus participate in the development of pro-atherogenic milieu. The effects induced by *H. pylori* components may be strengthened in the presence of 7-kCh and ASA. In this study, we verified this hypothesis using cell models of gastric epithelial cells and vascular endothelial cells, unexposed or pulsed in vitro with *H. pylori* soluble components alone or in combination with 7-kCh and ASA. We examined the ROS response of both cell types in conjunction with cell metabolic activity, apoptosis, and the permeability of cell monolayers. Furthermore, the secretion of proinflammatory interleukin (IL)-8, the signal transducer and activator of transcription 3 (STAT3) activation, and wound healing were evaluated. This study showed that the deleterious ROS response initiated by *H. pylori* in gastric epithelial cells and vascular endothelial cells was elevated in the presence of pro-atherogenic 7-kCh and the anti-aggregation drug ASA. Further studies are necessary to elucidate possible mechanisms driving the ROS-dependent deleterious effects. This knowledge may be important for medical practice in terms of diagnosing of patients with CHD for *H. pylori* infection and the eradication this infection in conjunction with using anti-aggregation drugs other than ASA.

## 2. Results

### 2.1. Upregulation of ROS Production in AGS or HUVEC Cell Cultures Carried out in the Presence of H. pylori Components Alone or Simultaneously with ASA and 7-kCh

**Figure 1:**
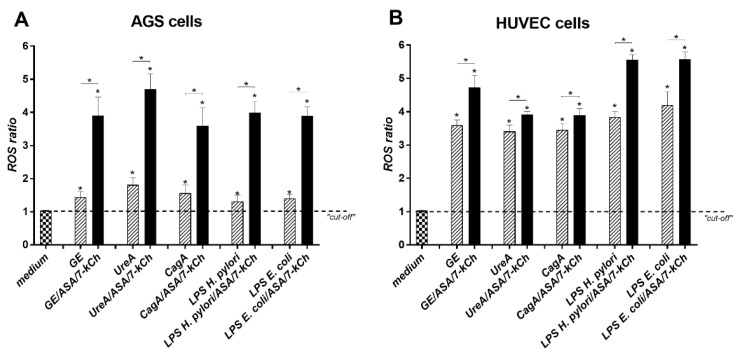
Reactive oxygen species. For the estimation of reactive oxygen species (ROS), the cell suspensions of gastric epithelial AGS cells (**A**) and vascular endothelial HUVEC cells (**B**) were treated for 24 h with *H. pylori* compounds: glycine acid extract—GE, subunit A of urease—UreA, cytotoxin associated gene A (CagA) protein, *H. pylori* lipopolysaccharide (LPS) or *E. coli* LPS, alone or in combination with acetylsalicylic acid—ASA, and 7-ketocholesterol—7-kCh, or in medium alone. The fluorescent probe—Dihydroetidine (DHE) was added to the wells, and fluorescence was measured. The ROS ratio was calculated based on relative fluorescence units (RFU) of stimulated cells vs. RFU of control cells in culture medium alone. Results are shown as means with standard deviations (SD) of five experiments performed in triplicates for each experimental variant. Statistical significance for * *p* < 0.05.

The components of *H. pylori*: GE, UreA, CagA, LPS, and the control *E. coli* LPS, significantly increased the amount of ROS in AGS and HUVEC cell cultures as compared to the unstimulated cells, which were propagated in a complete culture medium alone (Figure 1A,B). The HUVEC cells produced ROS upon induction with *H. pylori* components more effectively than AGS cells. In both cell lines exposed simultaneously to *H. pylori* components, ASA and 7-kCh, the ROS production was significantly higher than in cell cultures carried out in the presence to *H. pylori* components alone (Figure 1A,B).

### 2.2. The Correlation between Diminished Viability of AGS or HUVEC Cells and an Increased Number of Cells Undergoing Apoptosis in Cell Cultures Carried out with H. pylori Components Alone or in the Presence of ASA and 7-kCh

**Figure 2:**
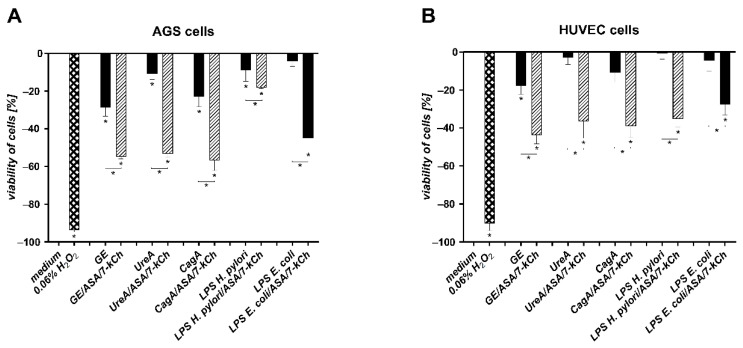
Assessment of cell viability. Gastric epithelial AGS cells (**A**) or vascular endothelial HUVEC cells (**B**) were sub-cultured for 24 h in the culture medium alone or exposed to *H. pylori* components: glycine acid extract—GE, subunit A of urease—UreA, cytotoxin associated gene A (CagA) protein, *H. pylori* lipopolysaccharide (LPS) or *E. coli* LPS alone or with acetylsalicylic acid—ASA, and 7-ketocholesterol—7-kCh. Cell viability was evaluated using the tetrazolium yellow dye MTT [3-(4,5-dimethylthiazol-2-yl)-2,5-diphenyltetrazolium bromide], which was reduced by living cells to yield soluble purple formazan crystals that were solubilized and detected colorimetrically. Results are presented as the percentage means ± standard deviation (SD) relative to untreated cells of at least four independent experiments performed in triplicates for each experimental variant. Statistical significance for * *p* < 0.05.

**Figure 3:**
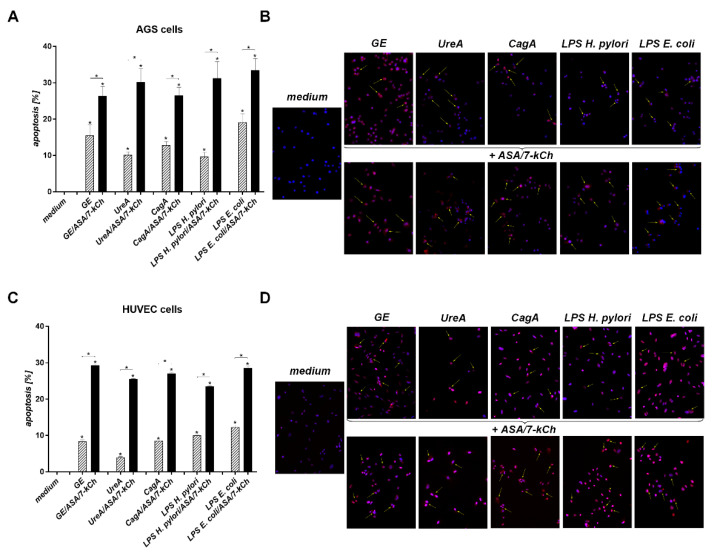
Assessment of cell apoptosis. Gastric epithelial AGS cells (**A**,**B**) or vascular endothelial HUVEC cells (**C**,**D**)) were sub-cultured in the culture medium alone or in the milieu of *H. pylori* components: glycine acid extract—GE, subunit A of urease—UreA, cytotoxin associated gene A (CagA) protein, *H. pylori* lipopolysaccharide (LPS) or *E. coli* LPS alone or with acetylsalicylic acid—ASA and 7-ketocholesterol—7-kCh. The intensity of AGS and HUVEC cell apoptosis was evaluated using terminal deoxynucleotidyl transferase dUTP nick end labeling (TUNEL) assay. Cell nuclei were counterstained with Hoechst. Cells with apoptotic changes (red) were imaged in the fluorescence microscope at magnification ×20. (**A**,**C**) The graphs indicate the percentage of apoptotic cells. (**B**,**D**) Representative images of cells stained in the TUNEL assay (red), at magnification ×20. The results of four independent experiments performed in triplicates for each experimental variant are presented. Statistical significance for * *p* < 0.05.

In the milieu of ROS, the risk of DNA damage increases, which may result in directing cells to apoptosis. We evaluated viability of AGS or HUVEC cells, and the number of cells undergoing apoptosis either unexposed or exposed to *H. pylori* components, in the presence or absence of ASA and 7-kCh, or in a culture medium alone, by the MTT reduction assay and the TUNEL assay, respectively (Figure 2 and Figure 3). The ability of AGS and HUVEC cells to reduce MTT was significantly diminished after the treatment of the cells with *H. pylori* components—GE, UreA, CagA, *H. pylori* LPS or *E. coli* LPS—alone vs control cells, which were sub-cultured in the complete culture medium. The number of cells which were able to reduce MTT was even lower in the cell cultures containing soluble *H. pylori* components as well as ASA and 7-kCh (Figure 2A,B). The treatment of AGS or HUVEC cells with *H. pylori* components resulted in an increased number of cells undergoing apoptosis compared to the unstimulated cells (Figure 3A–D). A further increase in the number of apoptotic cells was shown in both AGS or HUVEC cell cultures developed in the presence of the *H. pylori* components, ASA and 7-kCh. The increased number of metabolically inactive and apoptotic cells was combined with an elevated production of ROS in these cell cultures (Figure 2 and Figure 3).

### 2.3. Disintegration of AGS or HUVEC Cell Monolayers Treated with H. pylori Components Alone or Simultaneously with ASA and 7-kCh

**Figure 4:**
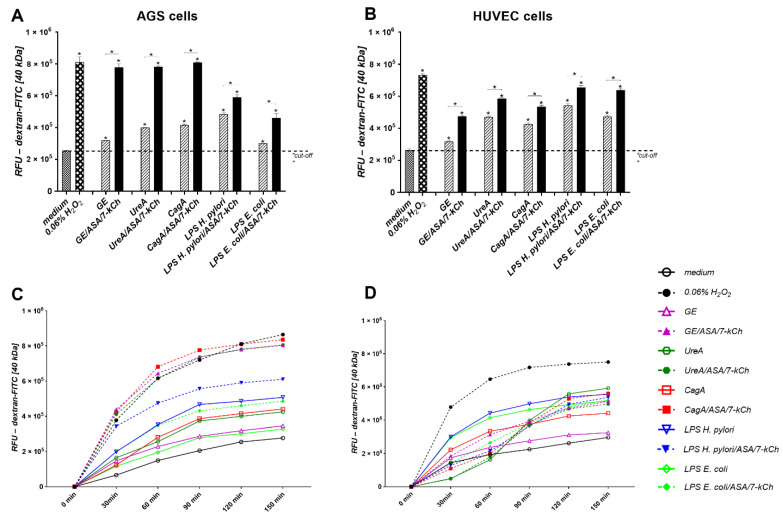
Permeability of cell monolayers—Paracellular flux assay. Gastric epithelial AGS cells (**A**) and vascular endothelial HUVEC cells (**B**) were cultured in the transwell system until they reached the confluence and then were treated with *H. pylori* components: glycine acid extract—GE, subunit A of urease—UreA, cytotoxin associated gene A (CagA) protein, *H. pylori* lipopolysaccharide (LPS) or *E. coli* LPS alone or with acetylsalicylic acid-ASA and 7-ketocholesterol—7-kCh. Thereafter, isothiocyanate fluorescein (FITC) dextran was added to the medium in the insert. The fluorescence of the FITC-dextran in the lower compartment was measured. The fluorescence intensity is shown as relative fluorescence units (RFU) after 120 min incubation of cells with dextran-FITC (**A**,**B** upper graphs) or at time 0 and after 30, 60, 90, 120, and 150 min of incubation (**C**,**D** lower graphs). Results are shown as means with standard deviation (SD) of four experiments performed in triplicates for each experimental variant. Statistical significance for * *p* < 0.05.

A decrease in the cell monolayer integrity due to an up-regulation of cell apoptosis should make the cellular barrier more permeable. We examined the influence of *H. pylori* components alone or in combination with ASA and 7-kCh on the permeability of the confluent monolayers of AGS and HUVEC cells. Unstimulated AGS or HUVEC cells were tight, whereas upon the induction with H_2_O_2_ the cell integrity was lost (positive control) (Figure 4). Similarly, the cell monolayers of AGS or HUVEC cells when treated for 120 min with *H. pylori* components—GE, UreA, CagA, LPS or *E. coli* LPS—passed more FITC-labeled dextran particles than the unstimulated cell monolayers. The supplementation of the culture medium containing the *H. pylori* components with ASA and 7-kCh resulted in the further reduction of cell integrity (Figure 4).

### 2.4. The Infuence of ASA or 7-kCh Alone on ROS Production, Cell Viability and Integrity, IL-8 Secretion, STAT3 Activation and Wound Healing in AGS or HUVEC Cell Models

**Figure 5:**
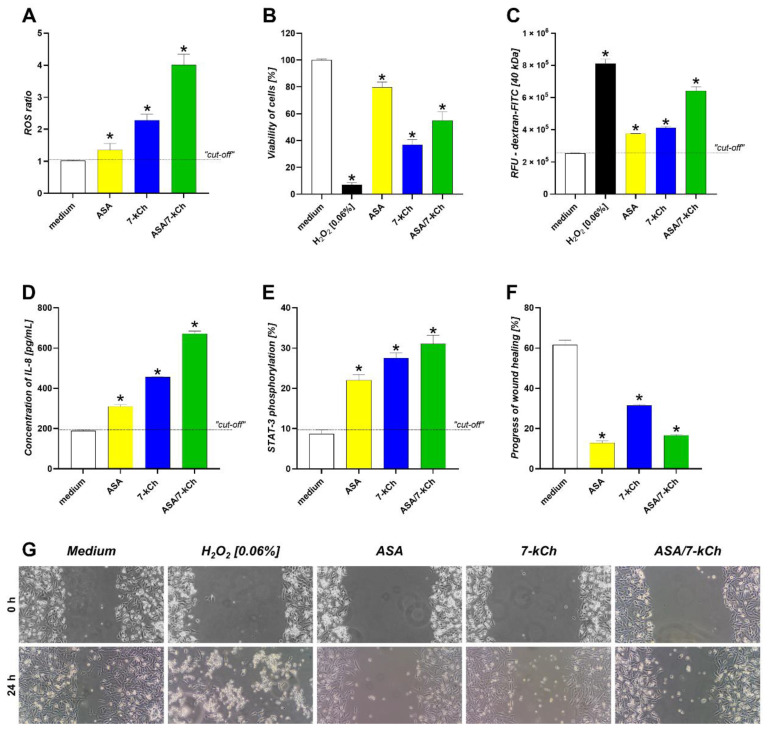
Effects of ASA and/or 7-kCh on gastric epithelial cells (AGS). AGS cells were treated for 24 h with acetylsalicylic acid (ASA) and/or 7-ketocholesterol (7-kCh), and then examined for: (**A**) reactive oxygen species (ROS); (**B**) cell viability; (**C**) permeability of cell monolayers for fluorescein isothiocyanate (FITC) bound dextran, expressed as relative fluorescence units (RFU); (**D**) the production of interleukin (IL)-8; (**E**) phosphorylation of signal transducer and activator of transcription 3 (STAT3). Progress of wound healing (**F**,**G**), was assessed in scratch assay. Results are shown as means with standard deviation (SD) of four experiments performed in triplicates for each experimental variant. Statistical significance for * *p* < 0.005. Cells in complete medium were used as control for natural cells. Cells treated with H_2_O_2_ (0.06%) were used as negative control in cell viability assay and paracellular flux assay.

In the AGS gastric epithelial cell cultures, the level of ROS was significantly increased after the exposure of the cells to ASA or 7-kCh alone compared to the control cells, which were grown only in a culture medium. The effects of ASA and 7-kCh on ROS production accumulated (Figure 5A). The elevated amounts of ROS correlated with the reduced viability of cells and increased permeability of the cell monolayers (Figure 5B,C). Furthermore, the disintegration of the cells exposed to ASA or 7-kCh, or both, resulted in a significant increase in the production of pro-inflammatory IL-8, in conjunction with the activation of the transcription factor STAT3 (Figure 5D,E). Moreover, the treatment of the gastric epithelial cells with ASA and/or 7-kCh delayed the wound healing process assessed in the scratch assay (Figure 5F).

**Figure 6:**
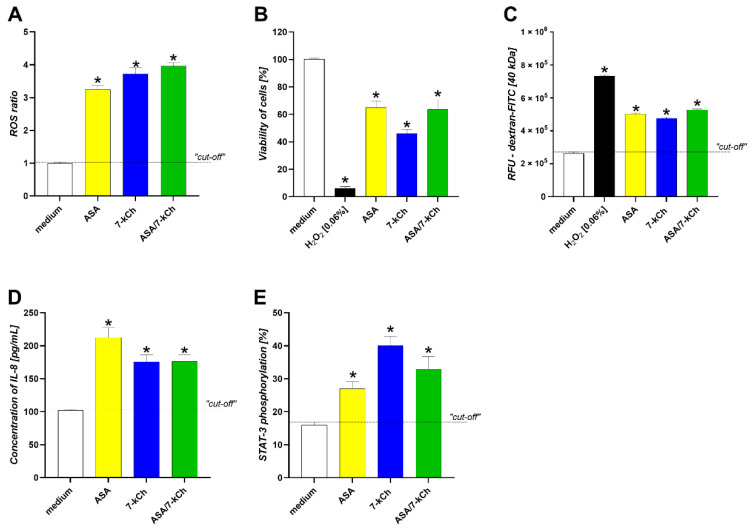
Effects of ASA and/or 7-kCh on vascular endothelial cells (HUVEC). HUVEC cells were treated 24 h with acetylsalicylic acid (ASA) and/or 7-ketocholesterol (7-kCh), and then examined for: (**A**) reactive oxygen species (ROS); (**B**) cell viability; (**C**) permeability of cell monolayers for fluorescein isothiocyanate (FITC) bound dextran, expressed as relative fluorescence units (RFU); (**D**) the production of interleukin (IL)-8; (**E**) phosphorylation of signal transducer and activator of transcription 3 (STAT3). Results are shown as means with standard deviation (SD) of four experiments performed in triplicates for each experimental variant. Statistical significance for * *p* < 0.005. RFU—Relative fluorescence units. Cells in complete culture medium (medium) were used as control for natural cells. Cells treated with H_2_O_2_ (0.06%) were used as negative control in cell viability assay and paracellular flux assay.

Similarly to the AGS cells, the HUVEC cells were sensitive to ASA and/or 7-kCh treatment, which was shown as the ROS elevation, the diminished cell viability and decreased cell to cell integrity, and the increased IL-8 production in conjunction with elevated STAT3 phosphorylation (Figure 6).

## 3. Discussion

*H. pylori* is an etiological agent of gastroduodenitis, gastric ulcers, and gastric cancer. The epidemiological and experimental data indicate the role of *H. pylori* infection plays in the development of systemic diseases, including atherosclerosis and coronary heart disease [5,12]. The dot-like colonization of the gastric mucosa by *H. pylori* may potentiate the damage and dysfunction of the gastric epithelial barrier due to the high local concentration of the soluble components of these bacteria. Mnich et al. (2016) [9], have suggested that the effects of *H. pylori* infection may depend on the particular component or released toxin, and their local concentrations. Virulence factors might be delivered by *H. pylori* actively or released due to bacterial cell lysis [9].

The role of *H. pylori* in the development of atherosclerosis and CHD is unclear. It has been shown that the majority of CHD patients are seropositive for anti-*H. pylori* antibodies, which confirms their exposure to these bacteria. Kowalski et al. (2001) [28] detected *H. pylori* DNA in atherosclerotic plaques, which means that these bacteria must have been present in the circulation, at least temporarily [28]. In addition, the presence of soluble immune complexes containing *H. pylori* antigens in the sera of CHD patients might reflect the systemic effects of *H. pylori* infection that are related to an increased inflammatory response in the vascular endothelium [13]. It has been shown that *Caviae porcellus* infected experimentally with *H. pylori* had more severe peripheral arterial stiffness compared to those non-infected [7].

In patients with CHD infected with *H. pylori*, changes in the gastric epithelium, and then in the vascular endothelium, may result from the combined effects of *H. pylori*’s components, endogenous lipids, and the cytotoxicity of the ASA used by CHD patients to prevent excessive blood clotting. Even in low doses, ASA can cause gastric tissue injury and thus drive a deleterious inflammatory response initiated by *H. pylori* [29]. ASA, by reducing the defensive functions of the gastric epithelium, may intensify the colonization of *H. pylori*. It has been suggested that in CHD patients infected with *H. pylori*, ASA may influence the development of a humoral response towards *H. pylori* antigens since in infected CHD patients the level of specific anti-*H. pylori* antibodies is even higher than in patients with *H. pylori* driven gastritis [14].

Potentially, oxidative stress induced during *H. pylori* infection may link this infection with an increased amount of endogenous 7-kCh which is a classic risk factor of CHD. Previously, we showed that the soluble components of or living *H. pylori* generate oxidative stress and induce cell apoptosis in the gastric tissue of *Caviae porcellus* infected with these bacteria. By using the cell cultures of the primary gastric epithelial cells and fibroblasts of these animals, it was revealed that an increased amount of ROS was followed by an elevated rate of apoptosis, an upregulation of both the local and systemic metalloproteinase (MMP)-9 concentrations, and diminished cell integrity [9,10]. Recently, Krupa et al. (2021) [30] showed that vascular endothelial cells are sensitive to *H. pylori* antigenic signals, which may initiate apoptosis in conjunction with an upregulation of MMP-9 [7]. It is possible that during infection with *H. pylori* both ROS and MMP-9-driven apoptosis may be involved in atherogenesis [30].

Epithelial barrier leakage may potentially facilitate the passage and systemic distribution of *H. pylori* components, particularly when the pro-regenerative activity of cells is diminished [10]. In the present study, we showed that the level of ROS in gastric epithelial cell cultures treated with *H. pylori* components—GE, UreA, CagA, or LPS—was significantly increased after the enrichment of the culture medium with 7-kCh and ASA. The elevated oxidative stress in these cell cultures was correlated with increased cell apoptosis and the disintegration of cell monolayers. Vascular endothelial cells also responded to *H. pylori* components alone, or in combination with ASA and 7-kCh, with ROS production, the upregulation of apoptosis, and a diminished cell to cell integrity. Thus, we asked whether ASA or 7-kCh alone could be a causative agent of these effects independently of *H. pylori* components. To answer this question, we developed the cell cultures of gastric epithelial cells or vascular endothelial cells using a complete culture medium enriched with ASA or 7-kCh separately, or in combination, without the addition of *H. pylori* components. In both types of cell cultures, the presence of ASA and/or 7-kCh increased the ROS level, which was combined with a diminished cell viability and cell integrity.

Cell death due to apoptosis protects tissues against harmful stress conditions, allowing the elimination of altered or abnormal cells. Apoptosis also controls the severity of inflammation [31]. However, the massive elimination of gastric epithelial cells due to *H. pylori* driven apoptosis may destabilize the gastric barrier, favoring the maintenance of the inflammatory response [32]. Another study also revealed that VacA *H. pylori* and outer inflammatory protein A (OipA) diminished the integrity of gastric epithelial cells due to apoptosis [33]. In the present study, we observed the progress of *H. pylori*-driven apoptosis of AGS and HUVEC cells in the milieu of ASA and 7-kCh. These two factors alone also showed pro-apoptotic activity towards these cells in vitro.

This study revealed that AGS and HUVEC cells were activated in response to ASA and/or 7-kCh as evidenced by the STAT3 phosphorylation and secretion of proinflammatory IL-8, which belongs to molecules that alert the immune system. In vivo, in *H. pylori* infected patients with a high fat diet-induced obesity, the expression of chemokines was significantly increased [34]. However, as we have demonstrated, the gastric epithelial cells were not able to migrate effectively within the scratch in the milieu of ASA and/or 7-kCh, which means that their potential for wound healing was diminished. A failure to return to homeostasis at the level of the gastric epithelium may result in the maintenance of pro-inflammatory stimulation.

The results obtained in this study indicate that *H. pylori* components, ASA or 7-kCh, independently, and particularly in combination, may contribute to adverse changes in the gastric epithelium and vascular endothelium by inducing excessive oxidative stress. In CHD patients infected with *H. pylori*, the deleterious effects of infection in the stomach, which are related to oxidative stress, can potentially accumulate in the presence of a classic risk factor like 7-kCh [35]. It has been shown that *H. pylori* upregulates the level of cholesterol, particularly the LDL fraction, and thus increased ROS may participate in the transformation of LDL to oxLDL, which can escalate the inflammatory processes in the vascular endothelium [36]. During *H. pylori* infection, ROS are delivered by gastric epithelial cells and immunocompetent cells which heavily infiltrate gastric mucosa. In the presence of oxLDL, macrophages infiltrating endothelial pro-atherogenic niches transform into foam cells, which are an integral part of the atherosclerotic plaque [35]. Such phenotypic changes in the macrophages can also be initiated directly by CagA-positive *H. pylori* strains or exosomal CagA derived from *H. pylori*-infected gastric epithelial cells as well as by LPS of these bacteria [7,37].

The proatherogenic activity of ROS also leads to an inactivation of nitric oxide and a modulation of redox-sensitive cell signaling pathways [38]. These may cause the dysfunction of the vascular–endothelial barrier, the development of a pathological inflammatory response, macrophage infiltration and activation, and the proliferation of smooth muscle cells that are involved in plaque formation [38]. Kiss et al. (2006) [39] showed that ROS modulate the activity of nuclear poly (ADP-ribose) polymerase (PARP), which may result in the reduction of the relaxing properties of the blood vessels [39]. Akbas et al. (2010) [40] revealed that in *H. pylori* infected subjects, the activity of serum paraoxygenase-1 is related to carotid intima media thickness [40].

Trachootham et al. (2008) [41] showed that moderate levels of ROS may function as signals promoting cell proliferation and survival, whereas in high concentrations ROS can induce cell death due to excessive DNA damage or the inhibition of DNA repair processes [41]. Kacprzak and Pawliczak (2015) [42] showed that aspirin-induced oxidative stress can be responsible for asthma exacerbation [42].

The side effects of ASA have been clinically recognized in CHD patients. Despite having a low risk of bleeding, nearly all patients receiving anti-platelet ASA therapy developed gastrointestinal injury [29]. Numerous studies are ongoing to assess the therapeutic efficacy of anticoagulants other than ASA [29,43,44]. For instance, among high bleeding-risk patients undergoing percutaneous intervention who completed a 3-month dual anti-platelet therapy without experiencing major adverse events, aspirin discontinuation followed by ticagrelor monotherapy significantly reduced bleeding without increasing ischemic events compared with ticagrelor plus aspirin [43].

The results obtained in this study indicate an accumulation of adverse effects in gastric epithelial cells and vascular endothelial cells in vitro in response to various *H. pylori* components, in the presence of 7-kCh, a classic CHD risk factor, and ASA, which is used as an anti-platelet drug in CHD therapy. These results suggest that *H. pylori* components together with 7-kCh and ASA may be involved in the maintenance of the inflammatory response during CHD. Due to the dot-like colonization of gastric mucosa by *H. pylori*, the concentration of different components of these bacteria may vary in the gastric mucosa and consequently some components may dominate at different time points during the infection. However, the cumulative effects of all *H. pylori* components are possible in vivo, which was shown in the current in vitro study. Updated in 2019, the data indicate several potential mechanisms linking *H. pylori* infection to the development of CHD [45].

Further studies are needed to find out whether the synergy effect of the components of *H. pylori*, 7-kCh, and ASA observed in this in vitro study of cell cultures of the gastric epithelial cells and vascular endothelial cells may take place in vivo.

## 4. Materials and Methods

### 4.1. Cell Cultures

Human AGS (CRL-1739) gastric adenocarcinoma epithelial cells from the American Type Culture Collection (ATCC, Rockville, MD, USA), were used in this study as they were previously [9]. These cells are often used in studying *H. pylori* pathogenic mechanisms in vitro. As immortalized cancer cells, AGS cells delivered a considerable number of cells at each passage. AGS cells were routinely grown as a monolayer in complete RPMI-1640 medium (cRPMI; Sigma-Aldrich Saint Louis, MI, USA), containing 10% heat inactivated Fetal Bovine Serum (FBS; CytoGen, Łódź, Poland), and 1% penicillin/streptomycin (Gibco, Zug, Switzerland), at 37 °C in a humidified atmosphere of a cell culture incubator containing 5% CO_2_. The cells were passaged with 0.25% trypsin in 0.02% ethylenediaminetetraacetic acid (EDTA) (Thermo Fisher Scientific, Waltham, MA, USA), every 7 days, and the medium was changed every 3–4 days. Primary human umbilical vein endothelial cells (HUVECs) (C2517A, Lonza, Walkersville, MD, USA), were expanded in Endothelial Basal Medium-2 (EGM-2) (Lonza, Walkersville, MD, USA), supplemented with EGM-2 BulletKit (Lonza, Walkersville, MD, USA). After reaching 80–90% confluence, HUVECs were trypsinized using 0.05% trypsin in the 0.02% EDTA (Sigma-Aldrich, Saint Louis, MO, USA), and neutralized by Trypsin Neutralizing Solution (Lonza, Walkersville, MD, USA). Both cell types were adjusted to appropriate density and used for further assays as described below.

### 4.2. Cell Stimulation

The reference *Helicobacter pylori* strain CCUG 17874 (Culture Collection, University of Gothenburg, Gothenburg, Sweden), positive for vacuolating toxin (VacA) and cytotoxin associated gene A (CagA) protein, was grown under microaerophilic conditions and bacterial surface components were extracted using 0.2M glycine buffer, pH 2.2, to obtain the glycine acid extract (GE). The GE has been dialyzed twice against PBS and evaluated for protein composition by sodium dodecyl sulphate polyacrylamide electrophoresis (SDS-PAGE) and Western blotting with the reference serum samples from *H. pylori* negative or *H. pylori* positive individuals (120 kDa, 80 kDa, between 66–42 kDa and 29–26 kDa) as previously described [9,46]. We used GE as a combination of different protein components of these bacteria, which might be released together with LPS from these bacteria during bacterial cell lysis in vivo. The protein content in GE was 600 μg/mL (NanoDrop 2000c Spectrophotometer, Thermo Fischer Scientific, Waltham, MA, USA). The GE sample contained <0.001 EU/mL of LPS as shown by the chromogenic Limulus amebocyte lysate test (Lonza, Walkersville, MD, USA). The UreA urease subunit (obtained by courtesy of M. Obuchowski and K. Hinc), was amplified by a polymerase chain reaction (PCR) as previously described [47]. Recombinant CagA protein (rCagA) was from IRIS, Siena, Italy (obtained by courtesy of A. Covacci). The protein was expressed in *E. coli* as a fusion protein as previously described [48]. LPS from the reference *H. pylori* strain CCUG 17874 (obtained by courtesy of AP. Moran), was prepared by hot phenol–water extraction, purified by proteinase K and RNA-se treatment and ultracentrifugation as previously described [49]. *E. coli* LPS derived from the O55:B5 strain (Sigma-Aldrich, Saint Louis, MO, USA), was used as control. The concentrations of *H. pylori* components and other stimulators for in vitro treatment of AGS or HUVEC cells were selected experimentally or used as previously described and were equal to: GE 10 µg/mL, UreA 5 µg/mL, CagA 1 µg/mL, *H. pylori* LPS and *E. coli* LPS 1 ng/mL, 7-kCh 20 µg/mL, and ASA 5 mM [9,10]. The stock solution of 7-kCh was prepared in 96% ethanol, and then diluted in PBS. The detrimental effect of the solvent (96% ethanol), diluted in PBS, has been excluded in the preliminary study. In all cell studies, all components were dissolved in a cell culture medium to obtain a safe dilution of the sample storage buffers. Cells were sub-cultured overnight in 24-well culture plates (1 × 10^6^ cells/well), in the RPMI-1640 complete culture medium, in the conditions of a cell culture incubator, to adhere, and then for further 24 h in the milieu of *H. pylori* components, with or without 7-kCh/ASA. Then, the culture supernatants and remaining cells were used for assessment of selected biomarkers and cell activity. Control cells were sub-cultured in cell culture medium alone (positive control of cell viability) or treated with 0.06% H_2_O_2_ (negative control of cell viability).

### 4.3. Assessment of Reactive Oxygen Species (ROS)

The induction of ROS in the AGS or HUVEC cell cultures was estimated according to Wojtala et al. (2014) [50]. For this purpose, cell suspensions in the culture medium (5 × 10^5^ cells/mL), were seeded in 96-well black plates for 24 h in cell culture incubator conditions. Further, they were treated for 24 h with *H. pylori* compounds, with or without 7-kCh/ASA, and then centrifuged (200× *g*, 10 min). The wells were emptied and supplemented with 200 µL 0.05 µM dihydroetidine (DHE, Sigma-Aldrich, Saint Louis, MO, USA), and after 20 min incubation (37 °C, 5% CO_2_), the cells were washed with phosphate buffered saline (PBS) and suspended in 5 mM glucose solution in PBS (200 µL/well). Fluorescence was measured by a Spectra Max i3 Platform^®^ reader (Molecular Devices, San Jose, CA, USA), at the following wavelengths: excitation 535 nm, emission 635 nm. The ROS ratio was calculated based on relative fluorescence units of stimulated cells (RFUs) versus relative fluorescence units of control cells in the cell culture medium alone (RFUu), according to the following formula: ROS ratio = RFUs/RFUu.

### 4.4. Cell Viability Assay

The cell viability of control cells, in culture medium alone, or in the milieu of stimulators, was evaluated by the ability of cells to reduce a tetrazolium yellow dye 3-(4,5-dimethylthiazol-2-yl)-2,5-diphenyltetrazolium bromide salt (MTT, Sigma, Saint Louis, MO, USA), according to the ISO norm 10993-5 (International Organization for Standardization, 2009), as previously described [9]. Absorbance at 570 nm was estimated with a plate reader Victor2 Wallac, Oy, Turku, Finland). The effectiveness of MTT reduction was calculated based on the following formula: MTT reduction relative to untreated cells (%) = (absorbance of treated cells/absorbance of untreated cells × 100%) − 100%.

### 4.5. Apoptosis

The AGS and HUVEC cells undergoing apoptosis were detected using the commercial terminal deoxynucleotidyl transferase dUTP nick end labeling (TUNEL) assay (Cell Meter TUNEL Apoptosis Assay Kit, AAT Bioques, Sunnyvale, CA, USA), as recommended by the manufacturer. Cells were treated with membrane-permeant fluorescent red dye that passively enters cells and selectively targets the nicks in DNA that form during apoptosis. Cell nuclei were counterstained with Hoechst (Sigma, Saint Louis, MO, USA) and diluted 1:1000 in PBS for 15 min at room temperature. Cells undergoing apoptosis (red), with fluorescently labeled DNA fragments, were imaged and photographed in the fluorescence microscope (Zeiss, Axio Scope, A1, Jena, Germany), at 550 nm excitation and 590 nm emission. The percentage of apoptotic cells was evaluated using the ImageJ Software version 1.48v (National Institute of Health, Bethesda, MD, USA), using the computer with Microsoft^®^ software Windows 8 (Redmond, WA, USA).

### 4.6. Cell Barrier Integrity—Paracelullar Flux Assay

Cells were seeded into 24-well plates in 6.5 mm transwell Culture Inserts (Greiner Bio-One, Kremsmünster, Austria), with 1.0 μm pore size in complete cell culture medium. After reaching confluence, the cells were stimulated for 24 h apically with *H. pylori* components: GE, UreA, CagA, *H. pylori* LPS or *E. coli* LPS alone or with such components in combination of ASA and 7-kCh. Control cells were propagated in complete culture medium or with ASA, or 7-kCh alone. Fluorescein isothiocyanate (FITC)-coupled dextran, molecular mass 40 kDa (FITC-dextran, Sigma-Aldrich, Saint Luis, MO, USA), was added to the upper compartment. The fluorescence of FITC-dextran in the lower compartment was measured in a Victor2 Microplate Reader (Wallac, Oy, Turku, Finland), at time 0 and after 30, 60, 90, 120, and 150 min of incubation, and expressed as relative fluorescence units (RFUs). The background fluorescence of cell culture medium was extracted from the experimental samples.

### 4.7. Wound Healing Assay

Cell migration was evaluated in vitro by a wound healing assay—Scratch assay, as previously described [9]. In brief, cells were seeded on 6-well plates at a density of 1 × 10^6^ cells/well in 1 mL cRPMI-1640, supplemented with 2% FCS and 1% penicillin and streptomycin (Sigma-Aldrich, Saint Louis, MO, USA), and cultured until complete confluence. The cell monolayers were scratched, and then incubated with or without tested compounds. Wound images were photographed after 24–48 h with a digital camera (Nikon P20, Tokyo, Japan) and saved in a tiff format. The scratched areas were measured using ImageJ software version 1.48v (National Institute of Health, Bethesda, MD, USA) in the computer with the Microsoft^®^ software Windows 8 (Redmond, WA, USA). Wound healing in cell cultures exposed to studied components was expressed as a percentage of cells migrating to the wound zone compared to that of the untreated cells. Four independent experiments were carried out with three replicates for each experimental variant.

### 4.8. STAT3 Activation

In response to cytokines and growth factors, STAT3 is phosphorylated and acts as transcription factor mediating the expression of a variety of genes, and thus plays a key role in many cellular processes, including cell growth and apoptosis. Activation of STAT3 was estimated using human/mouse phospho-STAT3 (Y705) immunoassay (R&D, Minneapolis, MN, USA), as recommended by the manufacturer. Both cell types, unstimulated (control) or stimulated for 24 h with studied components, were fixed with 4% formaldehyde solution for 10 min, washed 3 times in washing buffer, and subsequently stained with antibodies towards complete STAT3 or phosphorylated STAT3, using Spectra Max i3 Platform^®^ (Molecular Devices, San Jose, CA, USA), and then with the secondary antibodies labeled fluorescently. The level of fluorescence was estimated at 540/600 nm for phosphorylated STAT3 (pSTAT3) and at 360/450 nm for complete STAT3. The ratio of phosphorylated STAT3 to total STAT3 was determined by dividing the fluorescence of cells treated with anti-pSTAT3 antibodies by fluorescence of cells treated with anti-STAT3 antibodies according to the following formula: RFU pSTAT3/RFU STAT3. Three independent experiments were performed in triplicate.

### 4.9. IL-8 Production

Both cell types were incubated for 24 h in the complete culture medium alone or in the presence of tested stimulants. The concentration of IL-8 was determined in cell culture supernatants by the commercial enzyme linked immunosorbent assay (ELISA) with a sensitivity of 2 pg/mL (Invitrogen, TermoFisher Scientific, Waltham, MA, USA), as recommended by the manufacturer. Samples and IL-8 standard dilutions were added for binding to the wells of microplate coated with a target-specific antibodies. The sandwich was formed by the addition of biotinylated detection antibodies. Next, the solution of streptavidin-horseradish peroxidase complex was added to the wells, to deliver signal, which was evaluated colorimetrically using the microplate reader Victor 2 (Wallac, Oy, Turku, Finland).

### 4.10. Statistical Analysis

Data were expressed as the mean ± standard deviation (SD). The differences between groups were tested using the non-parametric Mann-Whitney U test. For statistical analysis, the GraphPad Prism 9.1 software (San Diego, CA, USA) was used. Results were considered statistically significant when *p* < 0.05.

## 5. Conclusions

The results of this in vitro study indicate that *H. pylori* components may affect the integrity of the gastric epithelial barrier due to increased oxidative stress and apoptosis, and these effects can potentially be further enhanced in the milieu of 7-kCh and ASA. A diminished gastric barrier integrity in vivo may enable the translocation of *H. pylori* components into the bloodstream, where these bacterial factors alone or under the influence of 7-kCh and ASA may cause microinjuries in the vascular endothelium due to elevation of ROS and apoptosis. This may facilitate the maintenance of the inflammatory response and the deposition of 7-kCh into the vascular endothelium. This cascade of events may potentially link *H. pylori* infection in CHD patients with the excessive inflammatory response and atherosclerosis processes. Further studies are necessary to determine the mechanisms for which components of *H. pylori* alone or accompanied by ASA and 7-kCh influence the vascular endothelium.

## Data Availability

Not applicable.

## Abbreviations

AGS	gastric adenocarcinoma epithelial cells
ASA	acetylsalicylic acid
ATCC	American Type Culture Collection
Bax	BCL2 associated x, apoptosis regulator
CagA	cytotoxin associated gene A antigen
CCUG	Culture Collection University of Gothenburg
CHD	coronary heart disease
MTT	3-(4,5-dimethylthiazol-2-yl)-2,5-diphenyltetrazolium bromide salt
c-Myc	Myc-proto-oncogene protein
DAPI	4′,6-diamidino-2-phenylindole
DHE	dihydroetidine
EDTA	ethylenediaminetetraacetic acid
EGM-2	endothelial growth medium-2
EU	European Unit
FBS	fetal bovine serum
FITC	fluorescein isothiocyanate
GE	glycine acid extract
GMP	guanosine monophosphate
HspB	heat shock protein B
HUVEC	human umbilical vein endothelial cells
7-kCh	7-ketocholesterol
LDL	low density lipoprotein
LPS	lipopolysaccharide
MAPK	mitogen-activated protein kinase
MMP-9	metalloproteinase 9
NFκB	nuclear factor kappa B
OipA	outer inflammatory protein A
oxLDL	oxidized low density lipoprotein
PARP	poly (ADP-ribose) polymerase
PBS	phosphate buffered saline
PCR	polymerase chain reaction
rCagA	recombinant cytotoxin associated gene A antigen
RFU	relative fluorescein unit
RPMI	Rosswell Park Memory Institute cell culture medium
ROS	reactive oxygen species
SD	standard deviation
SDS-PAGE	sodium dodecyl sulphate polyacrylamide gel electrophoresis
STAT3	signal transducer and activator of transcription 3
TLR-4	Toll-like receptor 4
TUNEL	terminal deoxynucleotidyl transferase dUTP nick end labelling
UreA	subunit A of urease
VacA	vacuolating cytotoxin A
